# Prebiotics to prevent necrotising enterocolitis in very preterm or very low birth weight infants

**DOI:** 10.1002/14651858.CD015133.pub2

**Published:** 2023-06-01

**Authors:** Sahar Sharif, Sam J Oddie, Paul T Heath, William McGuire

**Affiliations:** Centre for Reviews and DisseminationUniversity of YorkYorkUK; Bradford NeonatologyBradford Teaching Hospitals NHS Foundation TrustBradfordUK; Division of Child Health and Vaccine InstituteSt. George's, University of LondonLondonUK

**Keywords:** Humans, Infant, Newborn, Enterocolitis, Necrotizing, Enterocolitis, Necrotizing/etiology, Enterocolitis, Necrotizing/prevention & control, Infant, Extremely Premature, Infant, Premature, Diseases, Infant, Premature, Diseases/etiology, Infant, Premature, Diseases/prevention & control, Infant, Very Low Birth Weight, Infections

## Abstract

**Background:**

Dietary supplementation with prebiotic oligosaccharides to modulate the intestinal microbiome has been proposed as a strategy to reduce the risk of necrotising enterocolitis (NEC) and associated mortality and morbidity in very preterm or very low birth weight (VLBW) infants.

**Objectives:**

To assess the benefits and harms of enteral supplementation with prebiotics (versus placebo or no treatment) for preventing NEC and associated morbidity and mortality in very preterm or VLBW infants.

**Search methods:**

We searched the Cochrane Central Register of Controlled Trials (CENTRAL), MEDLINE, Embase, the Maternity and Infant Care database and the Cumulative Index to Nursing and Allied Health Literature (CINAHL), from the earliest records to July 2022. We searched clinical trials databases and conference proceedings, and examined the reference lists of retrieved articles.

**Selection criteria:**

We included randomised controlled trials (RCTs) and quasi‐RCTs comparing prebiotics with placebo or no prebiotics in very preterm (< 32 weeks' gestation) or VLBW (< 1500 g) infants. The primary outcomes were NEC and all‐cause mortality, and the secondary outcomes were late‐onset invasive infection, duration of hospitalisation since birth, and neurodevelopmental impairment.

**Data collection and analysis:**

Two review authors separately evaluated risk of bias of the trials, extracted data, and synthesised effect estimates using risk ratio (RR), risk difference (RD), and mean difference (MD), with associated 95% confidence intervals (CIs). The primary outcomes of interest were NEC and all‐cause mortality; our secondary outcome measures were late‐onset (> 48 hours after birth) invasive infection, duration of hospitalisation, and neurodevelopmental impairment. We used the GRADE approach to assess the level of certainty of the evidence.

**Main results:**

We included seven trials in which a total of 705 infants participated. All the trials were small (mean sample size 100). Lack of clarity on methods to conceal allocation and mask caregivers or investigators were potential sources of bias in three of the trials. The studied prebiotics were fructo‐ and galacto‐oligosaccharides, inulin, and lactulose, typically administered daily with enteral feeds during birth hospitalisation.

Meta‐analyses of data from seven trials (686 infants) suggest that prebiotics may result in little or no difference in NEC (RR 0.97, 95% CI 0.60 to 1.56; RD none fewer per 1000, 95% CI 50 fewer to 40 more; low‐certainty evidence), all‐cause mortality (RR 0.43, 95% CI 0.20 to 0.92; 40 per 1000 fewer, 95% CI 70 fewer to none fewer; low‐certainty evidence), or late‐onset invasive infection (RR 0.79, 95% CI 0.60 to 1.06; 50 per 1000 fewer, 95% CI 100 fewer to 10 more; low‐certainty evidence) prior to hospital discharge. The certainty of this evidence is low because of concerns about the risk of bias in some trials and the imprecision of the effect size estimates. The data available from one trial provided only very low‐certainty evidence about the effect of prebiotics on measures of neurodevelopmental impairment (Bayley Scales of Infant Development (BSID) Mental Development Index score < 85: RR 0.84, 95% CI 0.25 to 2.90; very low‐certainty evidence; BSID Psychomotor Development Index score < 85: RR 0.24, 95% 0.03 to 2.00; very low‐certainty evidence; cerebral palsy: RR 0.35, 95% CI 0.01 to 8.35; very low‐certainty evidence).

**Authors' conclusions:**

The available trial data provide low‐certainty evidence about the effects of prebiotics on the risk of NEC, all‐cause mortality before discharge, and invasive infection, and very low‐certainty evidence about the effect on neurodevelopmental impairment for very preterm or VLBW infants. Our confidence in the effect estimates is limited; the true effects may be substantially different. Large, high‐quality trials are needed to provide evidence of sufficient validity to inform policy and practice decisions.

## Plain language summary

**Prebiotics for preventing necrotising enterocolitis in preterm infants**

**Review question** Does giving very preterm or very low birth weight infants prebiotics prevent necrotising enterocolitis?

**Background** Very preterm (born more than eight weeks early) and very low birth weight (less than 1.5 kg) infants are at risk of developing necrotising enterocolitis, a severe condition where some lining of the infant's bowel becomes inflamed and dies. This condition is associated with death, serious infection, and long‐term disability and developmental problems. One way to help prevent necrotising enterocolitis may be to add prebiotics (non‐digestible sugar chains to support intestinal colonisation with healthy 'probiotic' bacteria) to milk feeds.

**What did we do?**

We searched for trials that looked examined the effect of prebiotics on the risk of necrotising enterocolitis in very preterm or very low birth weight infants. We compared and summarised the results of the trials and rated our confidence in the evidence, based on factors such as study methods and sizes.

**What did we find?** We found seven trials involving 705 infant participants in total. Trials were mostly small, and most had design flaws that might bias their findings.

**Key results** Combined analyses showed that giving very preterm or very low birth weight infants prebiotics may result in little or no difference in the risk of necrotising enterocolitis, death, or serious infection, but we have little confidence in the evidence. One trial assessed the effect on disability or developmental outcomes, but we have very little confidence in this evidence.

**What are the limitations of the evidence?** We have little confidence in the evidence for effects on necrotising enterocolitis, death, and serious infection (and very little confidence for the effect on disability or developmental outcomes) because of concerns that the methods used in the included trials may have introduced biases that exaggerated the benefits of prebiotics supplementation, and because some effect estimates are imprecise.

**How up to date is this evidence?**

The evidence is up to date to July 2022.

## Summary of findings

**Summary of findings 1:** Prebiotics compared to control in very preterm or very low birth weight infants

**Patient or population:** very preterm or very low birth weight infants **Setting:** neonatal care centres globally **Intervention:** prebiotics (fructo‐ and galacto‐oligosaccharides, inulin, lactulose) **Comparison:** control					
**Outcomes**	Anticipated absolute effects^*^ (95% CI)		**Risk ratio** **(95% CI)**	**№ of participants** **(trials)**	**Certainty of the evidence** **(GRADE)**
	**Risk with control**	**Risk with prebiotics**			
Necrotising enterocolitis (before hospital discharge)	86 per 1000	83 per 1000 (52 to 134)	0.97 (0.60 to 1.56)	686 (7)	⊕⊕⊝⊝ Low^a,b^
Mortality (all‐cause before hospital discharge)	25 per 1000	10 per 1000 (5 to 23)	0.43 (0.20 to 0.92)	686 (7)	⊕⊕⊝⊝ Low^a,b^
Late‐onset invasive infection (before hospital discharge)	237 per 1000	175 per 1000 (128 to 237)	0.79 (0.60 to 1.06)	686 (7)	⊕⊕⊝⊝ Low^a,b^
Bayley Scales of Infant Development Mental Development Index < 85 (assessed beyond infancy)	128 per 1000	108 per 1000 (32 to 372)	0.84 (0.25 to 2.90)	76 (1)	⊕⊝⊝⊝ Very low^c,d^
Bayley Scales of Infant Development Psychomotor Development Index < 85 (assessed beyond infancy)	121 per 1000	29 per 1000 (4 to 242)	0.24 (0.03 to 2.00)	68 (1)	⊕⊝⊝⊝ Very low^c,d^
Cerebral palsy (assessed beyond infancy)	26 per 1000	9 per 1000 (0 to 217)	0.35 (0.01 to 8.35)	76 (1)	⊕⊝⊝⊝ Very low^c,d^
*The risk in the intervention group (and its 95% confidence interval) is based on the assumed risk in the comparison group and the relative effect of the intervention (and its 95% CI). CI: confidence interval					
**GRADE Working Group grades of evidence** **High‐certainty:** we are very confident that the true effect lies close to that of the estimate of the effect. **Moderate‐certainty:** we are moderately confident in the effect estimate: the true effect is likely to be close to the estimate of the effect, but there is a possibility that it is substantially different. **Low‐certainty:** our confidence in the effect estimate is limited: the true effect may be substantially different from the estimate of the effect. **Very low‐certainty:** we have very little confidence in the effect estimate: the true effect is likely to be substantially different from the estimate of effect.					

^a^Downgraded one level for methodological limitations in two trials (risk of selection bias due to uncertainty about methods used to generate random sequence and conceal allocation), and with performance, detection, attrition, and reporting biases (one trial). ^b^Downgraded one level for imprecision of effect estimate (95% CI around estimate consistent with either benefit or harm/no effect). ^c^Downgraded one level for methodological limitations in one trial (risk of attrition bias). ^d^Downgraded two levels for serious imprecision of effect estimate (low number of participants and events).

## Background

This review assesses the trial evidence for the effectiveness of enteral supplementation with prebiotic oligosaccharides for preventing necrotising enterocolitis (NEC) in very preterm or very low birth weight (VLBW) infants. Other Cochrane Reviews intend to assess the evidence for prebiotics in combination with probiotics ('synbiotics') or probiotics alone (Sharif 2020; Sharif 2021).

### Description of the condition

Necrotising enterocolitis (NEC) is a syndrome of acute intestinal necrosis, which affects about one in 20 very preterm (born before 32 weeks' gestation) or VLBW (birth weight less than 1500 g) infants (Horbar 2012). The risk factors for NEC include being extremely preterm (born before 28 weeks' gestation) or extremely low birth weight (ELBW; birth weight less than 1000 g), and intrauterine growth restriction or compromise indicated by absent or reversed end‐diastolic flow velocities (AREDFV) in antenatal Doppler studies of the umbilical artery (Samuels 2017). Infants who develop NEC experience more episodes of severe infection, have lower levels of nutrient intake, grow more slowly, and have longer durations of hospital stay than gestation‐comparable infants who do not (Battersby 2018; Berrington 2012). The associated mortality rate is about 20%, and, in infants who survive NEC — especially if it is associated with bloodstream infections — there is a high risk of neurodevelopmental problems and disabilities (Hickey 2018).

The pathogenesis of NEC is incompletely understood, but intestinal dysbiosis, infection and inflammation are thought to contribute (Eaton 2017; Mara 2018; Stewart 2016). Evidence exists that the pattern, diversity and stability of the intestinal microbiome (microbial life and genes) is associated with the risk of developing NEC (Masi 2019; Olm 2019; Stewart 2012; Warner 2016). Feeding with human milk compared with cow‐milk formula reduces the risk of NEC in very preterm or VLBW infants (Cleminson 2015; Quigley 2019). One putative mechanism for this protective effect is that 'prebiotic' oligosaccharides, which are abundant in human milk (but not in standard formula), promote the growth of non‐pathogenic probiotic microorganisms, such as lactobacilli and bifidobacteria. These modulate the intestinal microbiome and enhance mucosal barrier functions (Embleton 2017; Granger 2020; Walsh 2019). Compared with human milk‐fed term infants, however, very preterm or VLBW infants tend to harbour fewer intestinal probiotic microorganisms, and more potential pathogens, which might be due to the dysbiotic effects of antibiotic exposure and enteral fasting during the early neonatal period (Stewart 2017).

### Description of the intervention

Prebiotics are a diverse family of complex glycans (chains of polymerised carbohydrates) that promote intestinal colonisation by probiotic microorganisms (Davani‐Davari 2019; Gibson 2017). Human milk contains numerous prebiotic substances, predominantly galacto‐oligosaccharides and fructo‐oligosaccharides (based on the sugars galactose and fructose, respectively), that influence the intestinal microbiome in preterm infants (Boehm 2008; Nolan 2020). More than 150 different prebiotic oligosaccharides have been detected in human milk, with about 20 of these accounting for almost all human milk oligosaccharide content in most women. The pattern of human milk oligosaccharides produced varies markedly between individual women, and can vary temporally (depending on the stage of lactation) within an individual woman (Austin 2019; Durham 2021; Smilowitz 2013).

Newborn infants do not digest human milk oligosaccharides. Rather, these are primarily nutrient sources for intestinal probiotic microorganisms, particularly bifidobacteria (Alcon‐Giner 2020; Jost 2015). Emerging evidence suggests that specific human‐milk oligosaccharides can promote probiotic predominance and reduce intestinal dysbiosis in very preterm infants (Masi 2021; Underwood 2015)*.* Manufactured or plant‐based (for example, inulin) prebiotic oligosaccharides are less heterogeneous than natural human‐milk oligosaccharides, typically consisting of chains of galactose or fructose, usually with a terminal glucose monomer (Johnson‐Henry 2016). These include lactulose, a non‐absorbable disaccharide synthesised from galactose and fructose (MacGillivray 1959). Evidence exists that giving supplemental, synthetic, prebiotic oligosaccharides to formula‐fed very preterm infants stimulates the growth of an intestinal microflora that is similar to that found in infants fed with maternal milk (Autran 2018; Boehm 2008; Kapiki 2007; Veereman‐Wauters 2011). Prebiotic oligosaccharides are added as ingredients to some cow‐milk formulas for feeding newborn infants for whom sufficient human milk is not available (Salminen 2020). Studies using animal models, however, have not provided consistent evidence of efficacy in preventing NEC‐like syndromes (Nolan 2020).

### How the intervention might work

The principal mechanism of action of supplemental prebiotics is likely to be the enhancement of probiotic microorganism growth and intestinal colonisation (Nolan 2020; Underwood 2019). Probiotic bacteria and fungi use prebiotic oligosaccharides as a major source of nutrients (Alcon‐Giner 2020). Promoting a probiotic‐rich intestinal microbiome is thought to benefit infants via several mechanisms. Probiotics may out‐compete pathogens for nutrients. Bifidobacteria and lactobacilli ferment prebiotic oligosaccharides to produce short‐chain fatty acids, including lactic acid, butyric acid, and propionic acid, that inhibit adhesion of pathogenic bacteria and modulate intestinal epithelial development, integrity, and barrier function (Johnson‐Henry 2016; Zmora 2018). Short‐chain fatty acids also lower the pH level of the stool and may enhance intestinal motility, thereby improving feed tolerance (Armanian 2019). Other putative actions include stimulating differentiation and proliferation of enterocytes (cells of the intestinal lining), enhancing expression of intestinal digestive enzymes, and improving intestinal mucosal barrier integrity (Johnson‐Henry 2016; Sanders 2019).

While there is some trial‐based evidence that enteral administration of exogenous probiotics reduces the risk of NEC and associated mortality and morbidity in very preterm or VLBW infants, concerns exist that effect size estimates are inflated by publication bias (Sharif 2020). Another major barrier to use of probiotic supplementation is uncertainty about the optimal constitution of products, as well as availability, and regulatory and licensing issues (Berrington 2019; Duffield 2019; Fleming 2019; Pell 2019; Vermeulen 2020). Furthermore, although existing data are reassuring with regard to safety, probiotic bacteraemia or fungaemia (the potentially problematic presence of live bacteria/fungi in the bloodstream) and other adverse effects have been reported in preterm infants (Bertelli 2015; Esaiassen 2016; Zbinden 2015).

### Why it is important to do this review

Necrotising enterocolitis and its associated complications — particularly invasive infection — are the commonest causes of mortality and serious morbidity beyond the early neonatal period in very preterm or VLBW infants (Berrington 2012). It is plausible that prebiotic supplementation might promote endogenous probiotic growth and colonisation, and reduce the risk of NEC and its associated morbidity and mortality (with fewer risks than exogenous probiotic supplementation). Appraising and synthesising the trial evidence about the effectiveness and safety of prebiotic supplementation could inform practice, policy and research.

## Objectives

To assess the benefits and harms of enteral supplementation with prebiotics (versus placebo or no treatment) for preventing NEC and associated morbidity and mortality in very preterm or VLBW infants.

## Methods

### Criteria for considering studies for this review

#### Types of studies

We included randomised or quasi‐randomised (predictable allocation) controlled trials, including cluster‐randomised controlled trials. Cross‐over studies were not eligible for inclusion.

#### Types of participants

Eligible participants were very preterm (< 32 weeks' gestation) or VLBW (< 1500 g) infants.

#### Types of interventions

The interventions of interest were prophylactic enteral prebiotics: any combination or dose of prebiotic oligosaccharides (galacto‐oligosaccharides (GOS); fructo‐oligosaccharides (FOS); inulin; or lactulose), commenced within 14 days of birth and continued (at least) daily for (at least) one week was eligible, versus placebo or no prebiotic.

We did not include trials of synbiotics (combinations of probiotics and prebiotics), or trials of other substances that may have some prebiotic properties, for example lactoferrin. The effectiveness of these interventions is addressed in other Cochrane Reviews (Pammi 2020; Sharif 2021).

#### Types of outcome measures

We focused on assessing effects on infant‐ and family‐important outcomes, principally neonatal morbidities that plausibly affect rates of mortality or neurodisability. We did not include surrogate outcomes such as stool colonisation patterns.

##### Primary outcomes

- NEC before discharge from hospital, confirmed at surgery or autopsy or using standardised clinical and radiological criteria (VON 2020):
  - at least one of: bilious gastric aspirate or emesis; or abdominal distention; or blood in stool; and
  - at least one of: abdominal radiograph showing pneumatosis intestinalis; or gas in the portal venous system; or free air in the abdomen
- All‐cause mortality before discharge from hospital

##### Secondary outcomes

- Late‐onset invasive infection, as determined by the culture of bacteria or fungus from blood or cerebrospinal fluid or from a normally sterile body space (> 48 hours after birth until discharge from hospital)
- Duration of hospitalisation since birth
- Neurodevelopmental impairment assessed by a validated test after 12 months' post‐term: neurological evaluations, developmental scores, and classifications of disability, including cerebral palsy and auditory and visual impairment

### Search methods for identification of studies

We used the criteria and standard methods of Cochrane Neonatal, as set out in our protocol (Sharif 2021).

#### Electronic searches

We searched the following electronic databases using a combination of text words and MeSH terms described in Appendix 1:

- Cochrane Central Register of Controlled Trials (CENTRAL; 2022, Issue 7), in the Cochrane Library;
- MEDLINE via Ovid (1946 to July 2022);
- Embase via Ovid (1974 to July 2022);
- Maternity & Infant Care Database via Ovid (1971 to June 2022);
- the Cumulative Index to Nursing and Allied Health Literature (CINAHL) (1982 to July 2022)

We limited the search outputs with filters for clinical trials as recommended in the *Cochrane Handbook for Systematic Reviews of Interventions* (Higgins 2020). We did not apply any language restrictions.

We searched clinical trials registries for ongoing or recently completed trials (clinicaltrials.gov; the World Health Organization's International Trial Registry Platform (www.who.int/clinical-trials-registry-platform), and the ISRCTN Registry (www.isrctn.com)).

#### Searching other resources

We examined the reference lists of any articles selected for inclusion in this review.

### Data collection and analysis

We used the standard methods of Cochrane Neonatal as set out in our protocol (Sharif 2021).

#### Selection of studies

Two review authors (SS and WM) independently screened the titles and abstracts of all studies and assessed the full articles for all potentially relevant trials. We excluded those reports that did not meet all the inclusion criteria, and we stated the reasons for exclusion. We discussed disagreements until consensus was achieved, with referral to a third author (SO or PTH) for final decision as necessary.

#### Data extraction and management

Two authors (SS, SO or WM) extracted data independently, using a form to aid extraction of information on design, methodology, participants, interventions, outcomes and treatment effects from each included study. We discussed disagreements until we reached a consensus. If data from the study reports were insufficient, we contacted the report authors for further information.

#### Assessment of risk of bias in included studies

Two review authors (SS, SO or WM) independently assessed the risk of bias (low, high or unclear) of all included trials using the Cochrane risk of bias tool (RoB 1) (Higgins 2011) for the following domains:

- sequence generation (selection bias);
- allocation concealment (selection bias);
- blinding of participants and personnel (performance bias);
- blinding of outcome assessment (detection bias);
- incomplete outcome data (attrition bias);
- selective reporting (reporting bias);
- any other bias (principally baseline imbalance).

Had any disagreements occurred, we planned to resolve these through discussion or by involving the third assessor. See Appendix 2 for a description of risk of bias for each domain. 

For cluster‐randomised trials, where groups of individuals rather than individuals were randomised to the different interventions, we additionally planned to assess bias arising from prior knowledge of cluster‐allocation (identification/recruitment bias, suggested by baseline imbalances in characteristics of participants rather than of clusters) and bias arising from the timing of identification and recruitment of participants (Higgins 2020).

#### Measures of treatment effect

We analysed the treatment effects in the individual trials and reported the risk ratio (RR) and risk difference (RD) for dichotomous data and the mean difference (MD) for continuous data, with respective 95% confidence intervals (CI). We planned to determine the number needed to treat for an additional beneficial outcome (NNTB) or an additional harmful outcome (NNTH) for analyses with a statistically significant difference in the RD.

#### Unit of analysis issues

The unit of analysis was the participating infant in individually randomized trials and the neonatal unit (or subunit) for cluster‐randomised trials. For cluster‐randomised trials, we planned to undertake analyses at the level of the individual while accounting for the clustering in the data using the methods recommended in the *Cochrane Handbook for Systematic Reviews of Interventions* (Higgins 2020).

#### Dealing with missing data

We planned to request additional data from trial investigators when data on important outcomes were missing or reported unclearly. If unavailable, we planned to undertake sensitivity analyses to assess the potential impact on outcomes by excluding those trials with > 20% missing data.

#### Assessment of heterogeneity

We examined the treatment effects of individual trials and heterogeneity between trial results by inspecting the forest plots. We calculated the I² statistic for each analysis to quantify inconsistency across studies and describe the percentage of variability in effect estimates that may be due to heterogeneity rather than to sampling error. If we detected high levels of heterogeneity (I² > 75%), we planned to explore the possible sources in subgroup analyses.

#### Assessment of reporting biases

If at least 10 trials were included in a meta‐analysis, we planned to examine a funnel plot for asymmetry visually and with Harbord's modification of Egger's test (Harbord 2006).

#### Data synthesis

We used a fixed‐effect inverse variance meta‐analysis for combining data where trials examined the same intervention and the populations and methods of the trials were judged to be similar.

#### Subgroup analysis and investigation of heterogeneity

When high heterogeneity was detected (I^2^ > 75%), we planned to examine the potential causes in subgroup analyses for the primary outcomes, specifically:

- type of prebiotic: GOS/FOS; inulin; or lactulose;
- type of enteral feeding permitted for participating infants: human milk, formula, or both;
- trials in which most (> 50%) participants were extremely low birth weight (ELBW; < 1000 g) or extremely preterm (< 28 weeks' gestation at birth) versus trials in which most infants were ≥ 28 weeks' gestation at birth or birth weight ≥ 1000 g;
- trials which restricted participation to infants with intrauterine growth restriction or absent or reversed end‐diastolic flow velocities in the foetal aorta or umbilical artery versus trials which did not do so.

#### Sensitivity analysis

We planned to undertake sensitivity analyses to determine how estimates are affected by including only studies at low risk of bias: (i) selection bias (adequate randomisation and allocation concealment), (ii) detection or performance bias (adequate masking of intervention and measurement), (iii) attrition bias (< 20% loss to follow‐up for primary outcome assessment), and (iv) reporting bias (selective reporting).

#### Summary of findings and assessment of the certainty of the evidence

Two authors (PTH, SO or WM) used the GRADE approach to assess the certainty of the evidence for effects on NEC, all‐cause mortality before hospital discharge, late‐onset invasive infection, and measures of neurodevelopmental impairment after 12 months' post‐term neurological evaluations, developmental scores, and classifications of disability, including cerebral palsy and auditory and visual impairment (Schünemann 2013; Walsh 2021).

We considered evidence from randomised controlled trials as high certainty but downgraded the evidence certainty by one level for serious (or two levels for very serious) limitations based upon the following domains: design (study limitations), inconsistency across studies, indirectness of the evidence, imprecision of estimates, and presence of publication bias. This approach results in an assessment of the certainty of a body of evidence as one of four grades:

- High certainty: further research is very unlikely to change our confidence in the estimate of effect.
- Moderate certainty: further research is likely to have an important impact on our confidence in the estimate of effect and may change the estimate.
- Low certainty: further research is very likely to have an important impact on our confidence in the estimate of effect and is likely to change the estimate.
- Very low certainty: we are very uncertain about the estimate.

We used GRADEpro GDT to create summary of findings table and to report the certainty of the evidence.

## Results

### Description of studies

#### Results of the search

After the removal of duplicates from the search results, we screened 3282 titles and abstracts. We evaluated 14 articles sourced as full‐text reports (Figure 1) and of these, we included seven studies. No ongoing studies were identified.

**1:**
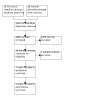
Study flow diagram

#### Included studies

See: Characteristics of included studies.

We included seven trials (Armanian 2014; Boehm 2002; Dilli 2015; Hascoët 2022; Modi 2010; Riskin 2010; van den Berg 2010). Most were conducted during the past 20 years, predominantly in Europe (five trials), as well as Iran (one trial) and Israel (one trial). Most trials were performed in single centres; three were multicentre trials (Dilli 2015; Hascoët 2022; Modi 2010). In all the trials, individual infants were allocated randomly to intervention or control groups. None used a cluster design.

##### Population

In total, 705 infants participated in the included trials (mean 100). Three trials enrolled only very preterm or VLBW infants. Four trials enrolled infants of gestational age up to 32 weeks', and because the average gestation at birth was < 32 weeks', or the average birth weight < 1500 g, we included these trials (Boehm 2002; Hascoët 2022; Modi 2010; Riskin 2010). One trial excluded infants who were born with birth weight below the 10th percentile for the reference population ("small‐for‐gestation") (Modi 2010). None of the trials specified exclusion of infants who had evidence of absent or reversed end‐diastolic flow velocities detected on antenatal Doppler studies of the foetal aorta or umbilical artery.

In most trials, participating infants were permitted human milk or formula feeding. One trial enrolled infants who received human milk only (Armanian 2014), and one trial enrolled only formula‐fed participants (Boehm 2002).

##### Interventions and comparisons

The prebiotic preparations tested varied. Four trials used short‐chain galacto‐oligosaccharides/long‐chain fructo‐oligosaccharides (9:1 ratio) (Armanian 2014; Boehm 2002; Modi 2010; van den Berg 2010), with one trial additionally including 20% pectin‐derived acidic oligosaccharides (van den Berg 2010). One trial used inulin, a plant fructan (Dilli 2015), one used lactulose, a synthetic fructose‐galactose disaccharide (Riskin 2010), and one used a combination of two human milk oligosaccharides; 2′‐fucosyllactose (2′FL) and lacto‐N‐neotetraose (LNnT) in a 10:1 ratio (Hascoët 2022). These were mostly commercially‐available products supplied by the manufacturer for use in the trial. Six trials were placebo‐controlled (maltodextrin or glucose).

Most trials started prebiotic (and placebo if used) supplements during the first week after birth when enteral feeding with human milk or formula was tolerated. In five of the trials, prebiotics or placebo were administered daily until discharge from hospital (Armanian 2014; Dilli 2015; Hascoët 2022; Modi 2010; Riskin 2010). In two trials the intervention was continued for four weeks (Boehm 2002; van den Berg 2010).

##### Outcomes

All the trials reported the number of infants who developed NEC, all‐cause mortality, and late‐onset invasive infection. In one trial, none of the participants experienced any of these outcomes (Boehm 2002). Other in‐hospital outcomes reported included time to establish full enteral feeding, rate of weight gain, and duration of hospital stay. Only one of the trials reported neurodevelopmental outcomes (van den Berg 2010).

#### Excluded studies

See Characteristics of excluded studies. We excluded seven reports of studies. Six trials were excluded because participants were term or late‐preterm (not very preterm) infants (Dasopoulou 2015; Fanaro 2005; Indrio 2009; Kapiki 2007; Luoto 2013; Neumer 2021). One trial was excluded because participating infants did not commence supplements until beyond the neonatal period, when the risk of the outcomes for this review occurring was already much reduced (Mihatsch 2006).

### Risk of bias in included studies

Risk of bias assessments and judgements are described in Characteristics of included studies and are summarised in Figure 2.

**2:**
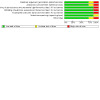
Risk of bias graph: review authors' judgements about each risk of bias item presented as percentages across all included studies

#### Allocation

Most trial reports described methods to generate random sequences (typically computer‐generated) and to ensure adequate allocation concealment (typically sealed opaque envelopes). One report did not describe the methods used to randomise infants (Boehm 2002). One trial was quasi‐randomised (high risk) with treatment allocation based on the infant's case file number (Armanian 2014).

#### Blinding

Six trials were placebo‐controlled (Boehm 2002; Dilli 2015; Hascoët 2022; Modi 2010; Riskin 2010; van den Berg 2010). The other trial did not mask parents, caregivers, or clinical investigators (Armanian 2014).

#### Incomplete outcome data

Six trials reported complete or near‐complete assessments of primary outcomes (Boehm 2002; Dilli 2015; Hascoët 2022; Modi 2010; Riskin 2010; van den Berg 2010). In one trial, primary outcome data were not available for more than one‐quarter of participants in the intervention group (Armanian 2014).

#### Selective reporting

Although trial protocols were not available for most trials, selective reporting bias was not considered a major threat given that all relevant clinical outcomes were reported.

#### Other potential sources of bias

We did not find evidence of between‐group baseline differences in participant characteristics or demographics in six trials (Armanian 2014; Boehm 2002; Dilli 2015; Hascoët 2022; Modi 2010; van den Berg 2010). In one trial, the mean birth weight and gestational age differed substantially between the groups (Riskin 2010). These differences were not explained in the report.

### Effects of interventions

See: Table 1

#### Primary outcomes

##### NEC

Meta‐analysis of data from seven trials (686 infants) suggests that prebiotics may result in little or no difference in NEC prior to hospital discharge (Analysis 1.1; Figure 3):

**3:**
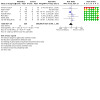
Forest plot: effects of prebiotics versus control on necrotising enterocolitis

- RR 0.97, 95% CI 0.60 to 1.56;
- RD none fewer per 1000, 95% CI 50 fewer to 40 more.

###### Subgroup analysis for heterogeneity

In the absence of high levels of heterogeneity (I² = 0%), we did not undertake subgroup analyses (Subgroup analysis and investigation of heterogeneity).

Using the GRADE approach, we assessed the certainty of the evidence to be 'low'. We downgraded evidence certainty by one level for study limitations and one level for imprecision of the effect estimate (Table 1).

##### All‐cause mortality before hospital discharge

Meta‐analysis of data from seven trials (686 infants) suggests that prebiotics may result in little or no difference in all‐cause mortality prior to hospital discharge (Analysis 1.2; Figure 4):

**4:**
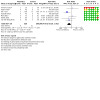
Forest plot: effects of prebiotics versus control on all‐cause mortality

- RR 0.43, 95% CI 0.20 to 0.92;
- RD 40 per 1000 fewer, 95% CI 70 fewer to none fewer.

###### Subgroup analysis for heterogeneity

In the absence of high levels of heterogeneity (I² = 22%), we did not undertake subgroup analyses (Subgroup analysis and investigation of heterogeneity).

We assessed the certainty of evidence to be 'low'. We downgraded evidence certainty by one level for study limitations and by one level for imprecision (Table 1).

#### Secondary outcomes

##### Late‐onset invasive infection

Meta‐analysis of data from seven trials (686 infants) suggests that prebiotics may result in little or no difference in late‐onset invasive infection prior to hospital discharge (Analysis 1.3; Figure 5):

**5:**
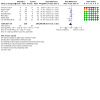
Forest plot: effects of prebiotics versus control on late‐onset invasive infection

- RR 0.79, 95% CI 0.60 to 1.06;
- RD 50 per 1000 fewer, 95% CI 100 fewer to 10 more.

###### Subgroup analysis for heterogeneity

In the absence of high levels of heterogeneity (I² = 0%), we did not undertake subgroup analyses (Subgroup analysis and investigation of heterogeneity).

We assessed the certainty of evidence to be 'low'. We downgraded evidence certainty by one level for study limitations and by one level for imprecision (Table 1).

##### Duration of hospitalisation

Two trials reported a shorter median duration of hospitalisation with prebiotics versus control:

- Armanian 2014: 16 versus 25 days*;
- Dilli 2015: 38 versus 50 days*.

Two trials did not report a difference:

- Riskin 2010: 53 versus 72 days*;
- van den Berg 2010: 52 versus 54 days*.

Three trials did not report duration of hospitalisation (Boehm 2002; Hascoët 2022; Modi 2010).

*Meta‐analysis was not possible as standard errors were not reported.

##### Neurodevelopmental impairment

One trial assessed neurodevelopmental impairment in surviving children at the corrected age of two years (van den Berg 2010). Outcomes were assessed in 76 infants (75% of eligible participants).

The evidence if very uncertain about the effect of prebiotics on the median Bayley Scales of Infant Development (second or third edition) Index scores:

- Mental Development Index (MDI): 95 (range 80 to 115) versus 100 (range 65 to 115);
- Psychomotor Development Index (PDI): 100 (range 71 to 130) versus 97 (range 69 to 145).

The evidence is very uncertain about the effect of prebiotics on the proportion of infants with Bayley Scales of Infant Development MDI score < 85 (indicative of developmental delay) (Analysis 1.4):

- RR 0.84, 95% CI 0.25 to 2.90;
- RD 20 per 1000 fewer, 95% CI 170 fewer to 120 more.

The evidence is very uncertain about the effect of prebiotics on the proportion of infants with Bayley Scales of Infant Development PDI scores < 85 (Analysis 1.5):

- RR 0.24, 95% 0.03 to 2.00;
- RD 90 per 1000 fewer, 95% CI 220 fewer to 30 more.

The evidence is very uncertain about the effect of prebiotics on the proportion of infants diagnosed with cerebral palsy (Analysis 1.6):

- RR 0.35, 95% CI 0.01 to 8.35;
- RD 30 per 1000 fewer, 95% CI 90 fewer to 40 more.

None of the infants had auditory and visual impairment.

We assessed the certainty of evidence to be 'very low' because of study limitations and serious imprecision of effect estimate (Table 1).

#### Sensitivity analyses of trials at low risk of bias

We undertook sensitivity meta‐analyses of data from the three trials (467 infants) at low risk of bias across all domains (Dilli 2015; Modi 2010; van den Berg 2010). These showed similar results compared with the primary analyses:

- NEC: RR 0.98, 95% CI 0.58 to 1.65; RD 0 per 1000 fewer, 95% 60 fewer to 50 more (Analysis 2.1);
- All‐cause mortality prior to hospital discharge: RR 0.38, 95% CI 0.17 to 0.89; RD 50 per 1000 fewer, 95% 90 to 10 fewer (Analysis 2.2);
- Late‐onset invasive infection: RR 0.82, 95% CI 0.59 to1.14; RD 40 per 1000 fewer, 95% 110 fewer to 30 more (Analysis 2.3);
- Neurodevelopmental outcomes: as above; data from van den Berg 2010 only (Analysis 2.4; Analysis 2.5; Analysis 2.6).

## Discussion

### Summary of main results

Meta‐analyses of data from seven trials suggests that enteral supplementation with prebiotics may result in little or no difference in NEC, all‐cause mortality, or late‐onset invasive infection prior to hospital discharge, but the evidence is of low certainty. Four trials reported a shorter median duration of hospitalisation with prebiotics versus control, and two trials did not show a difference. These trials did not provide data to permit meta‐analysis. Only one trial assessed neurodevelopmental impairment and the evidence of effect is of very low certainty.

### Overall completeness and applicability of evidence

These data are likely to be relevant to current practice since all the included trials were conducted during the past 25 years in neonatal care facilities across a variety of settings (Iran, Germany, Turkey, England, Israel, Netherlands, France). The risk of developing NEC amongst infants in both the control and intervention groups was about 5% to 10%, similar to incidence estimates from recent observational studies (Battersby 2018; Horbar 2012). While most participants were very preterm or VLBW infants, few were extremely preterm or ELBW. However, only one of the trials specifically excluded infants born 'small for gestational age' (Modi 2010). None excluded infants who had evidence of absent or reversed end‐diastolic flow velocities in antenatal Doppler studies of the umbilical artery or foetal aorta, increasing the applicability of the review findings to these populations of very preterm of VLBW infants at high risk of NEC and associated mortality and morbidity.

The trials used a variety of prebiotics. The most commonly assessed formulations were plant‐derived and synthetic galacto‐oligosaccharides and fructo‐oligosaccharides constituted to mimic oligosaccharides found in human milk (Armanian 2014; Boehm 2002; Dilli 2015; Modi 2010; van den Berg 2010). One trial assessed lactulose, a synthetic fructose‐galactose disaccharide (Riskin 2010). Only one trial assessed human milk oligosaccharides (2′‐fucosyllactose and lacto‐*N*‐neotetraose) (Hascoët 2022). These were mostly commercially‐available products supplied by the manufacturer for use in the trial. A better understanding of the mechanisms and events occurring at the intestinal epithelial and mucosal level may help to determine which prebiotics optimally supports a putatively beneficial microbiome in very preterm or VLBW infants (Abbas 2021; Autran 2018).

The type of enteral feeds that infants receive might influence the effects of prebiotic supplementation (Quigley 2019). One trial permitted only human milk feeding, two trials recruited formula‐fed infants, while in the other three trials infants could be fed with human milk or formula, or both. In the absence of high levels of heterogeneity, we did not undertake any subgroup analyses by type of milk feeding. Any such analysis, furthermore, would need to be interpreted cautiously as the data available were insufficient to define subgroups at an infant (rather than trial) level. The possibility remains that infants who receive human milk as their predominant source of nutrition might not gain added benefit from prebiotics supplementation since their milk is already rich in human milk oligosaccharides that enhance probiotic growth and colonisation (Nolan 2020).

### Quality of the evidence

We used GRADE methods to assess the certainty of the evidence for effects on NEC, all‐cause mortality, late‐onset invasive infection, and neurodevelopmental impairment (Table 1). We downgraded the certainty of the evidence because of methodological weaknesses (risk of bias) in three of the trials (Armanian 2014; Boehm 2002; Riskin 2010). These included uncertainty about measures to conceal allocation and to mask parents, caregivers, and clinical assessors that may have introduced selection, performance and detection biases. In one trial, there was unexplained baseline imbalance with the mean gestational age and birth weight higher in intervention than control groups. A priori, therefore, infants in the intervention group were at lower average risk than control infants of NEC, all‐cause mortality, late‐onset invasive infection, and neurodevelopmental impairment, potentially leading to over‐estimates of effect sizes. However, prespecified sensitivity analyses of the three trials (467 infants) at low risk of bias across all domains showed effects consistent with those in the primary meta‐analyses that included all the trials (Dilli 2015; Modi 2010; van den Berg 2010).

The other reason for downgrading the certainty of the evidence was the existence of substantial imprecision in estimates of effect, with meta‐analyses generating 95% CI that included large benefit as well as small or no benefit or harm. Estimates of effect were imprecise, especially for less common outcomes, including all‐cause mortality prior to hospital discharge, where the 95% CI ranged from an NNTB from 80 fewer to none fewer per 1000 infants given prebiotic supplements. Such imprecise estimates of effect are unlikely to meaningfully inform decision‐making in this context.

### Potential biases in the review process

We used the standard methods of Cochrane Neonatal to minimise potential biases in the review process. Two authors performed the literature search independently and combined results. We contacted study investigators to clarify inclusion criteria where necessary, and to provide unpublished data and missing information. Following full‐text screening, we excluded six studies because of the characteristics of their participant populations (term or near‐term infants rather than very preterm infants). We made one marginal decision to exclude another study on the grounds that participating very preterm infants commenced prebiotic supplements only when fully‐fed; mean day 36 for prebiotics, day 53 for maltodextrin placebo (Mihatsch 2006). Although this was not a prespecified exclusion criterion, we agreed that the study differed substantially from the review's intent, that is, focused primarily on preventing necrotising enterocolitis.

An important concern with the review process is the possibility that the findings are subject to publication and other reporting biases. Data from trials which show statistically significant or potentially important effects tend to be more readily available for inclusion in meta‐analyses (Gale 2020). Publication bias, as well as other sources of small‐study bias, is an important contributor to inflation of effect size estimates in meta‐analyses of interventions to improve outcomes in very preterm or VLBW infants (Young 2021). For example, the Cochrane Review of probiotics to prevent NEC in very preterm or VLBW infants showed a large reduction in the risk of NEC, but the funnel plot and regression analysis indicated that publication bias was likely to have inflated the pooled effect size estimate (Sharif 2020). In this review, we could not assess whether publication bias (or related small study biases) exaggerated the effect size since the meta‐analyses contained insufficient data points (fewer than 10) to make funnel plot inspection and regression analysis valid and reliable; that is, able to distinguish real asymmetry from chance asymmetry (Higgins 2020). Although we attempted to minimise the threat of publication bias by screening the reference lists of included trials and related reviews and searching the proceedings of the major international perinatal conferences to identify trial reports that are not published in full form in academic journals, we cannot be sure that other trials have been undertaken but not reported.

### Agreements and disagreements with other studies or reviews

We are aware of one other systematic review that assessed the trial evidence for prebiotics supplementation in preterm infants (Srinivasjois 2013). Although this review employed less stringent inclusion criteria than our review has (for example, including trials in which term infants participated), the findings were similar, that is, suggesting that prebiotic supplementation has little or no effect on the risk of NEC or associated morbidity.

Other Cochrane Reviews have addressed whether probiotics alone or synbiotics (probiotics combined with prebiotics) affect the risk of NEC (Sharif 2020; Sharif 2021). Meta‐analyses of data from trials of probiotic or synbiotics supplementation suggested a reduction in the risk of NEC and associated morbidity and all‐cause mortality for very preterm or VLBW infants. Similar to the findings in this review, however, concerns about trial quality, heterogeneity of interventions, imprecision, and publication bias, as well as the paucity of data for extremely preterm or ELBW infants, means that these findings are of low certainty, and should be interpreted and applied cautiously.

## Authors' conclusions

**Implications for practice**

The available trial data provide low‐certainty evidence about the effects of prebiotics on the risk of necrotising enterocolitis (NEC), all‐cause mortality before discharge, and invasive infection, and very low‐certainty evidence about the effect on neurodevelopmental impairment, for very preterm or very low birth weight (VLBW) infants. Our confidence in the effect estimates is limited; further research is very likely to have an important impact on the estimates of effect. In addition to concern about biases in the existing trials, a major barrier to implementing the findings is that existing analyses are not able to determine reliably the optimal constitution of prebiotic supplements (as well as doses, timing of introduction, duration of use) for routine prophylactic use. A variety of commercially available prebiotic preparations are in use in a minority of neonatal units internationally, but widespread use is limited by availability and regulatory and licensing issues.

**Implications for research**

Given the low level of certainty about whether (and which) prebiotics affect important outcomes in very preterm or VLBW infants, further high‐quality randomised, placebo‐controlled trials are needed to provide evidence of sufficient validity to inform policy and practice. Such trials are likely to need to recruit several thousands of infant participants to reliably detect plausible effects on uncommon outcomes such as NEC and mortality prior to hospital discharge (Gale 2020). Ideally, trials should attempt to ensure that caregivers and assessors are masked to the intervention, as investigation and diagnosis of NEC, late‐onset invasive infection and neurodevelopmental impairment can be subjective and can be associated with the inter‐rater variation. While it may be appropriate to be broadly inclusive of very preterm and VLBW infant participants, trials should ensure sufficient power to assess effects in extremely preterm or extremely low birth weight (ELBW) infants, infants born 'small for gestational age', or with evidence of absent or reversed end‐diastolic flow velocities in antenatal Doppler studies of the umbilical artery or foetal aorta. Trials, furthermore, should be powered to explore interactions with the type of enteral feed (human milk versus cow‐milk formula) received (Quigley 2019). Investigators need to consider which types of prebiotic to evaluate in trials, including perhaps those specific human milk oligosaccharides that have been associated with a lower risk of NEC in preterm infants (Masi 2021), and whether trials using prebiotics are merited alongside trials of probiotics and synbiotics as part of a factorial or an adaptive design (Underwood 2019).

## History

Protocol first published: Issue 8, 2021

## Appendices

### Appendix 1. Search strategies

#### Electronic databases

Search date: 5 July 2022

##### Cochrane Register of Controlled Trials (CENTRAL)

#1 [mh Probiotics]

#2 (probiotic*):ti,ab,kw

#3 [mh Bifidobacterium]

#4 (bifidobacterium*):ti,ab,kw

#5 [mh Lactobacillus]

#6 (lactobacill*):ti,ab,kw

#7 ([mh ^Saccharomyces] or [mh ^"Saccharomyces boulardii"] or [mh ^"Saccharomyces cerevisiae"])

#8 [mh ^"Saccharomyces boulardii"]

#9 (Saccharomyces):ti,ab,kw

#10 #1 OR #2 OR #3 OR #4 OR #5 OR #6 OR #7 OR #8 OR #9

#11 [mh Prebiotics]

#12 (prebiotic*):ti,ab,kw

#13 [mh Oligosaccharides]

#14 (oligosaccharide*):ti,ab,kw

#15 [mh Inulin]

#16 (inulin*):ti,ab,kw

#17 ((fructooligosaccharide* or fructo NEXT oligosaccharide* or FOS or FOSs or galacto NEXT oligosaccharide* or galactooligosaccharide*)):ti,ab,kw

#18 [mh Lactoferrin]

#19 (lactoferrin*):ti,ab,kw

#20 [mh Lactulose] 439

#21 (lactulose*):ti,ab,kw

#22 #11 OR #12 OR #13 OR #14 OR #15 OR #16 OR #17 OR #18 OR #19 or #20 or #21

#23 [mh Synbiotics]

#24 (synbiotic*):ti,ab,kw

#25 (((probiotic* and prebiotic*) NEAR/4 combin*)):ti,ab,kw

#26 #23 OR #24 OR #25

#27 #10 OR #22 OR #26

#28 [mh "Infant, Newborn"]

#29 [mh "Premature Birth"]

#30 neonat*:ti,ab,kw

#31 neo NEXT nat*:ti,ab,kw

#32 newborn or new NEXT born* or newly NEXT born*:ti,ab,kw

#33 preterm or preterms or pre NEXT term or pre NEXT terms:ti,ab,kw

#34 preemie* or premie or premies:ti,ab,kw

#35 prematur* NEAR/3 (birth* or born or deliver*):ti,ab,kw

#36 low NEAR/3 (birthweight* or birth NEXT weight*):ti,ab,kw

#37 lbw or vlbw or elbw:ti,ab,kw

#38 infan* or baby or babies:ti,ab,kw

#39 #28 or #29 or #30 or #31 or #32 or #33 or #34 or #35 or #36 or #37 or #38

#40 #27 AND #39 in Trials

##### CINAHL via EBSCO

S35 S31 AND S34

S34 S32 OR S33

S33 TX ( (neonat* or neo nat*) ) OR TX ( (newborn* or new born* or newly born*) ) OR TX ( (preterm or preterms or pre term or pre terms) ) OR TX ( (preemie$ or premie or premies) ) OR TX ( (prematur* N3 (birth* or born or deliver*)) ) OR TX ( (low N3 (birthweight* or birth weight*)) ) OR TX ( (lbw or vlbw or elbw) ) OR TX infan* OR TX ( (baby or babies) )

S32 (MH "Infant, Newborn+")

S31 S22 AND S30

S30 S28 not S29

S29 ( MH animals+ OR MH (animal studies) OR TI (animal model*) ) NOT MH (human) 194,413

S28 S23 OR S24 OR S25 OR S26 OR S27

S27 AB (cluster W3 RCT)

S26 MH placebos OR PT randomized controlled trial OR AB control W5 group OR MH crossover design OR MH comparative studies

S25 MH sample size AND AB ( (assigned OR allocated OR control) )

S24 TI ( (randomised OR randomized) ) OR AB random* OR TI trial

S23 MH Randomized Controlled Trials OR MH double‐blind studies OR MH single‐blind studies OR MH random assignment OR MH pretest‐posttest design OR MH cluster sample

S22 S9 OR S18 OR S21

S21 S19 OR S20

S20 TI ( (probiotic* and prebiotic*) N4 combin* ) OR AB ( (probiotic* and prebiotic*) N4 combin* )

S19 TI Synbiotic* OR AB Synbiotic*

S18 S10 OR S11 OR S12 OR S13 OR S14 OR S15 OR S16 OR S17

S17 TI Lactoferrin OR AB Lactoferrin

S16 TI fructooligosaccharide* OR AB fructooligosaccharide* OR TI fructo‐oligosaccharide* OR AB fructo‐oligosaccharide* OR TI galactooligosaccharide* OR AB galactooligosaccharide* OR TI galacto‐oligosaccharide* OR AB galacto‐oligosaccharide*

S15 TI Inulin OR AB Inulin

S14 TI lactulose* OR AB lactulose*

S13 TI Oligosaccharides OR AB Oligosaccharides

S12 MH "Oligosaccharides"

S11 TI Prebiotic* OR AB Prebiotic*

S10 MH "Prebiotics"

S9 S1 OR S2 OR S3 OR S4 OR S5 OR S6 OR S7 OR S8

S8 TI Saccharomyces OR AB Saccharomyces

S7 MH "Saccharomyces"

S6 TI lactobacillus OR AB lactobacillus

S5 (MH "Lactobacillus") OR (MH "Lactobacillus Acidophilus")

S4 TI bifidobacterium* OR AB bifidobacterium*

S3 MH "Bifidobacterium"

S2 TI probiotic* OR AB probiotic*

S1 MH "Probiotics"

##### Embase via Ovid <1974 to 1 July 2022>

1 Probiotic Agent/

2 probiotic$.ti,ab,kw.

3 exp bifidobacterium/

4 bifidobacterium$.ti,ab,kw.

5 exp lactobacillus/

6 lactobacill$.ti,ab,kw.

7 Saccharomyces/ or Saccharomyces boulardii/ or Saccharomyces cerevisiae/

8 Saccharomyces$.ti,ab,kw.

9 or/1‐8

10 Prebiotic Agent/

11 prebiotic$.ti,ab,kw.

12 exp Oligosaccharide/

13 oligosaccharide$.ti,ab,kw.

14 Galactose oligosaccharide/

15 (galacto‐oligosaccharide$ or galactooligosaccharide$).ti,ab,kw.

16 Fructose Oligosaccharide/

17 (fructooligosaccharide$ or fructo‐oligosaccharide$ or FOS or FOSs).ti,ab,kw.

18 Lactulose/

19 lactulose$.ti,ab,kw.

20 Inulin/

21 inulin$.ti,ab,kw.

22 Lactoferrin/

23 lactoferrin$.ti,ab,kw.

24 or/10‐23

25 Synbiotic Agent/

26 synbiotic$.ti,ab,kw.

27 ((probiotic$ and prebiotic$) adj4 combin$).ti,ab,kw.

28 25 or 26 or 27

29 9 or 24 or 28

30 Newborn/

31 Prematurity/

32 (neonat$ or neo nat$).ti,ab.

33 (newborn$ or new born$ or newly born$).ti,ab.

34 (preterm or preterms or pre term or pre terms).ti,ab.

35 (preemie$ or premie or premies).ti,ab.

36 (prematur$ adj3 (birth$ or born or deliver$)).ti,ab.

37 (low adj3 (birthweight$ or birth weight$)).ti,ab.

38 (lbw or vlbw or elbw).ti,ab.

39 infan$.ti,ab.

40 (baby or babies).ti,ab.

41 or/30‐40

42 Randomized controlled trial/

43 Controlled clinical study/

44 Random$.ti,ab.

45 randomization/

46 intermethod comparison/

47 placebo.ti,ab.

48 (compare or compared or comparison).ti.

49 ((evaluated or evaluate or evaluating or assessed or assess) and (compare or compared or comparing or comparison)).ab.

50 (open adj label).ti,ab.

51 ((double or single or doubly or singly) adj (blind or blinded or blindly)).ti,ab.

52 double blind procedure/

53 parallel group$1.ti,ab.

54 (crossover or cross over).ti,ab.

55 ((assign$ or match or matched or allocation) adj5 (alternate or group$1 or intervention$1 or patient$1 or subject$1 or participant$1)).ti,ab.

56 (assigned or allocated).ti,ab.

57 (controlled adj7 (study or design or trial)).ti,ab.

58 (volunteer or volunteers).ti,ab.

59 human experiment/

60 trial.ti.

61 or/42‐60

62 (random$ adj sampl$ adj7 ("cross section$" or questionnaire$1 or survey$ or database$1)).ti,ab. not (comparative study/ or controlled study/ or randomi?ed controlled.ti,ab. or randomly assigned.ti,ab.)

63 Cross‐sectional study/ not (randomized controlled trial/ or controlled clinical study/ or controlled study/ or randomi?ed controlled.ti,ab. or control group$1.ti,ab.)

64 (((case adj control$) and random$) not randomi?ed controlled).ti,ab.

65 (Systematic review not (trial or study)).ti.

66 (nonrandom$ not random$).ti,ab.

67 "Random field$".ti,ab.

68 (random cluster adj3 sampl$).ti,ab.

69 (review.ab. and review.pt.) not trial.ti.

70 "we searched".ab. and (review.ti. or review.pt.)

71 "update review".ab.

72 (databases adj4 searched).ab.

73 (rat or rats or mouse or mice or swine or porcine or murine or sheep or lambs or pigs or piglets or rabbit or rabbits or cat or cats or dog or dogs or cattle or bovine or monkey or monkeys or trout or marmoset$1).ti. and animal experiment/

74 Animal experiment/ not (human experiment/ or human/)

75 or/62‐74

76 61 not 75

77 29 and 41 and 76

##### Maternity & Infant Care Database (MIDIRS) via OVID <1971 to 14 June 2022>

1 probiotic$.ti,ab,de.

2 bifidobacterium$.ti,ab,de.

3 lactobacill$.ti,ab,de.

4 Saccharomyces$.ti,ab,de.

5 or/1‐4

6 prebiotic$.ti,ab,de.

7 oligosaccharide$.ti,ab,de.

8 inulin$.ti,ab,de.

9 (fructooligosaccharide$ or fructo‐oligosaccharide$ or FOS or FOSs).ti,ab,de.

10 (galactooligosaccharide$ or galacto‐oligosaccharide$).ti,ab,de.

11 lactoferrin$.ti,ab,de.

12 lactulose$.ti,ab,de.

13 or/6‐12

14 synbiotic$.ti,ab,de.

15 ((probiotic$ and prebiotic$) adj4 combin$).ti,ab,de.

16 14 or 15

17 5 or 13 or 16

18 (neonat$ or neo nat$).ti,ab.

19 (newborn$ or new born$ or newly born$).ti,ab.

20 (preterm or preterms or pre term or pre terms).ti,ab.

21 (preemie$ or premie or premies).ti,ab.

22 (prematur$ adj3 (birth$ or born or deliver$)).ti,ab.

23 (low adj3 (birthweight$ or birth weight$)).ti,ab.

24 (lbw or vlbw or elbw).ti,ab.

25 infan$.ti,ab.

26 (baby or babies).ti,ab.

27 or/18‐26

28 17 and 27

29 limit 28 to randomised controlled trial

##### Ovid MEDLINE(R) ALL <1946 to 1 July 2022>

1 Probiotics/

2 probiotic$.ti,ab,kw.

3 exp bifidobacterium/

4 bifidobacterium$.ti,ab,kw.

5 exp lactobacillus/

6 lactobacill$.ti,ab,kw.

7 Saccharomyces/ or Saccharomyces boulardii/ or Saccharomyces cerevisiae/

8 Saccharomyces$.ti,ab,kw.

9 or/1‐8

10 Prebiotics/

11 prebiotic$.ti,ab,kw.

12 Oligosaccharides/

13 oligosaccharide$.ti,ab,kw.

14 (galactooligosaccharides or galacto‐oligosaccharides).ti,ab,kw.

15 (fructooligosaccharide$ or fructo‐oligosaccharide$ or FOS or FOSs).ti,ab,kw.

16 Lactulose/

17 lactulose$.ti,ab,kw.

18 Inulin/

19 inulin$.ti,ab,kw.

20 Lactoferrin/

21 lactoferrin$.ti,ab,kw.

22 or/10‐21

23 Synbiotics/

24 synbiotic$.ti,ab,kw.

25 ((probiotic$ and prebiotic$) adj4 combin$).ti,ab,kw. (374)

26 or/23‐25

27 9 or 22 or 26

28 exp Infant, Newborn/

29 Premature Birth/

30 (neonat$ or neo nat$).ti,ab.

31 (newborn$ or new born$ or newly born$).ti,ab.

32 (preterm or preterms or pre term or pre terms).ti,ab.

33 (preemie$ or premie or premies).ti,ab.

34 (prematur$ adj3 (birth$ or born or deliver$)).ti,ab.

35 (low adj3 (birthweight$ or birth weight$)).ti,ab.

36 (lbw or vlbw or elbw).ti,ab.

37 infan$.ti,ab.

38 (baby or babies).ti,ab.

39 or/28‐38

40 randomized controlled trial.pt.

41 controlled clinical trial.pt.

42 randomized.ab.

43 placebo.ab.

44 drug therapy.fs.

45 randomly.ab.

46 trial.ab.

47 groups.ab.

48 or/40‐47

49 exp animals/ not humans.sh.

50 48 not 49

51 27 and 39 and 50

‐‐‐‐‐‐‐‐‐‐‐‐‐‐‐‐‐‐‐‐‐‐‐‐‐‐‐‐‐‐‐‐‐‐‐‐‐‐‐‐‐‐‐‐‐‐‐‐‐‐‐‐‐‐‐‐‐‐‐‐‐‐‐‐‐‐‐‐‐‐‐‐‐‐‐‐‐‐‐‐

#### Trials registers

Search date: 5 July 2022

##### WHO ICTRP *via trialsearch.who.int/*

Search 1 of 2:  Condition: (infant* OR baby OR babies OR premature or neonate* OR new born OR preterm OR low birth weight OR low birthweight OR LBW OR VLBW or ELBW) AND Intervention: (probiotic* OR bifidobacterium OR lactobacillus OR saccharomyces OR prebiotic* OR oligosaccharide* OR galactooligosaccharide* OR galacto‐oligosaccharide*) Recruitment Status: ALL

Search 2 of 2:  Condition: (infant* OR baby OR babies OR premature or neonate* OR new born OR preterm OR low birth weight OR low birthweight OR LBW OR VLBW or ELBW) AND Intervention: (fructooligosaccharide* OR fructo‐oligosaccharide* OR FOS OR lactulose OR inulin OR lactoferrin OR synbiotics) Recruitment Status: ALL

##### Clinical Trials.gov via clinicaltrials.gov/

Search 1 of 2:  Other terms: (infant OR baby OR premature OR neonate OR "new born" OR preterm OR "low birth weight" OR LBW OR VLBW OR ELBW) AND (probiotics OR bifidobacterium OR lactobacillus OR saccharomyces OR prebiotics OR oligosaccharides OR galactooligosaccharides)

Search 2 of 2:  Other terms: (infant OR baby OR premature OR neonate OR "new born" OR preterm OR "low birth weight" OR LBW OR VLBW OR ELBW) AND (fructooligosaccharide OR fos OR lactulose OR inulin OR lactoferrin OR synbiotics)

### Appendix 2. Risk of bias tool

#### *Sequence generation (checking for possible selection bias). Was the allocation sequence adequately generated?*

For each included study, we categorised the method used to generate the allocation sequence as:

- low risk (any truly random process, e.g. random number table; computer random number generator);
- high risk (any non‐random process, e.g. odd or even date of birth; hospital or clinic record number); or
- unclear risk.

#### *Allocation concealment (checking for possible selection bias). Was allocation adequately concealed?*

For each included study, we categorised the method used to conceal the allocation sequence as:

- low risk (e.g. telephone or central randomisation; consecutively numbered sealed opaque envelopes);
- high risk (open random allocation; unsealed or non‐opaque envelopes, alternation; date of birth); or
- unclear risk.

#### *Blinding of participants and personnel (checking for possible performance bias). Was knowledge of the allocated intervention adequately prevented during the study?*

For each included study, we categorised the methods used to blind study participants and personnel from knowledge of which intervention a participant received. Blinding was assessed separately for different outcomes or class of outcomes. We categorised the methods as:

- low risk, high risk or unclear risk for participants; and
- low risk, high risk or unclear risk for personnel.

#### *Blinding of outcome assessment (checking for possible detection bias). Was knowledge of the allocated intervention adequately prevented at the time of outcome assessment?*

For each included study, we categorised the methods used to blind outcome assessment. Blinding was assessed separately for different outcomes or class of outcomes. We categorised the methods as:

- low risk for outcome assessors;
- high risk for outcome assessors; or
- unclear risk for outcome assessors.

#### *Incomplete outcome data (checking for possible attrition bias through withdrawals, dropouts, protocol deviations). Were incomplete outcome data adequately addressed?*

For each included study and for each outcome, we described the completeness of data including attrition and exclusions from the analysis. We noted whether attrition and exclusions were reported, the numbers included in the analysis at each stage (compared with the total randomised participants), reasons for attrition or exclusion where reported, and whether missing data were balanced across groups or were related to outcomes. Where sufficient information was reported or supplied by the trial authors, we re‐included missing data in the analyses. We categorised the methods as:

- low risk (< 20% missing data);
- high risk (≥ 20% missing data); or
- unclear risk.

#### *Selective reporting bias. Are reports of the study free of suggestion of selective outcome reporting?*

For each included study, we described how we investigated the possibility of selective outcome reporting bias and what we found. For studies in which study protocols were published in advance, we planned to compare prespecified outcomes versus outcomes eventually reported in the published results. If the study protocol was not published in advance, we planned to contact study authors to gain access to the study protocol. We assessed the methods as:

- low risk (where it is clear that all of the study's prespecified outcomes and all expected outcomes of interest to the review have been reported);
- high risk (where not all the study's prespecified outcomes have been reported; one or more reported primary outcomes were not prespecified outcomes of interest and are reported incompletely and so cannot be used; study fails to include results of a key outcome that would have been expected to have been reported); or
- unclear risk.

#### *Other sources of bias. Was the study apparently free of other problems that could put it at a high risk of bias?*

For each included study, we described any important concerns we had about other possible sources of bias (for example, whether there was a potential source of bias related to the specific study design or whether the trial was stopped early due to some data‐dependent process). We assessed whether each study was free of other problems that could put it at risk of bias as:

- low risk;
- high risk;
- unclear risk.

If needed, we planned to explore the impact of the level of bias through undertaking sensitivity analyses.

## Data and analyses

**Comparison 1:** Prebiotics versus control

Outcome or subgroup title	No. of studies	No. of participants	Statistical method	Effect size
1.1 Necrotising enterocolitis	7	686	Risk Ratio (M‐H, Fixed, 95% CI)	0.97 [0.60, 1.56]
1.2 All‐cause mortality	7	686	Risk Ratio (M‐H, Fixed, 95% CI)	0.43 [0.20, 0.92]
1.3 Late‐onset invasive infection	7	686	Risk Difference (M‐H, Fixed, 95% CI)	‐0.05 [‐0.10, 0.01]
1.4 Bayley Scales of Infant Development Mental Development Index < 85	1	76	Risk Difference (M‐H, Fixed, 95% CI)	‐0.02 [‐0.17, 0.12]
1.5 Bayley Scales of Infant Development Psychomotor Development Index < 85	1	68	Risk Difference (M‐H, Fixed, 95% CI)	‐0.09 [‐0.22, 0.03]
1.6 Cerebral palsy	1	76	Risk Difference (M‐H, Fixed, 95% CI)	‐0.03 [‐0.09, 0.04]

**Comparison 2:** Prebiotics versus control (trials at low risk of bias)

Outcome or subgroup title	No. of studies	No. of participants	Statistical method	Effect size
2.1 Necrotising enterocolitis	3	467	Risk Ratio (M‐H, Fixed, 95% CI)	0.98 [0.58, 1.65]
2.2 All cause mortality	3	467	Risk Ratio (M‐H, Fixed, 95% CI)	0.38 [0.17, 0.89]
2.3 Late‐onset invasive infection	3	467	Risk Difference (M‐H, Fixed, 95% CI)	‐0.04 [‐0.11, 0.03]
2.4 Bayley Scales of Infant Development Mental Development Index < 85	1		Risk Ratio (M‐H, Fixed, 95% CI)	Totals not selected
2.5 Bayley Scales of Infant Development Psychomotor Development Index < 85	1		Risk Ratio (M‐H, Fixed, 95% CI)	Totals not selected
2.6 Cerebral palsy	1		Risk Ratio (M‐H, Fixed, 95% CI)	Totals not selected

## Characteristics of studies

### Characteristics of included studies [ordered by study ID]

**Table:** Armanian 2014

***Study characteristics***		
Methods	RCT	
Participants	88 VLBW infants (gestational age < 35 weeks') ‐ human milk fed	
Interventions	Prebiotics (N = 34): GOS/FOS (9:1 mixture: 0.5 to 1.5 g/kg) added to human milk daily until hospital discharge Control (N = 54): no prebiotic supplement	
Outcomes	NEC Death Invasive infection Feed intolerance Length of hospital stay	
Notes	Setting: Iran (2012 to 2013) Funding: Nutricia MMP, Mashhad, Iran	
***Risk of bias***		
**Bias**	**Authors' judgement**	**Support for judgement**
Random sequence generation (selection bias)	High risk	"infants into two groups based on their file number" "unequal randomization" (1:2)
Allocation concealment (selection bias)	High risk	"those with an even digit at the end of their file numbers were placed in [prebiotic] group and neonates with their file numbers ending in an odd digit were assigned to [control] group"
Blinding of participants and personnel (performance bias) All outcomes	High risk	Unmasked
Blinding of outcome assessment (detection bias) All outcomes	High risk	Unmasked
Incomplete outcome data (attrition bias) All outcomes	High risk	Outcome data not available for 15 infants (26% of intervention group, 7% of control group)
Selective reporting (reporting bias)	Low risk	Unlikely
Other bias	Low risk	No evidence of baseline imbalance

**Table:** Boehm 2002

***Study characteristics***		
Methods	RCT	
Participants	30 formula‐fed preterm infants < 33 weeks' gestation*	
Interventions	Prebiotics (N = 15): GOS/FOS (9:1 mixture: 1 g/100 mL) mixed with preterm formula for 28 days Control (N = 15): maltodextrin placebo	
Outcomes	Stool colonisation patterns Stool frequency and consistency Weight gain [No episodes of NEC, all cause mortality, or invasive infection]	
Notes	Setting: Germany (late 1990s) Funding: Numico Research (Germany) *Mean gestational age 31 weeks Infants commenced supplements when fully formula‐fed (mean day 8 for both groups)	
***Risk of bias***		
**Bias**	**Authors' judgement**	**Support for judgement**
Random sequence generation (selection bias)	Unclear risk	"infants were randomly assigned"
Allocation concealment (selection bias)	Unclear risk	"infants were randomly assigned"
Blinding of participants and personnel (performance bias) All outcomes	Low risk	Masked ("compositions of the two formulas were, apart from the supplemented oligosaccharides, identical")
Blinding of outcome assessment (detection bias) All outcomes	Low risk	Masked ("compositions of the two formulas were, apart from the supplemented oligosaccharides, identical")
Incomplete outcome data (attrition bias) All outcomes	Low risk	Complete reporting
Selective reporting (reporting bias)	Unclear risk	Clinical outcomes not reported
Other bias	Low risk	No evidence of baseline imbalance

**Table:** Dilli 2015

***Study characteristics***		
Methods	RCT	
Participants	200 very preterm or VLBW infants	
Interventions	Prebiotics (N = 100): inulin (900 mg) added to human milk or formula once daily for 8 weeks (or until hospital discharge) Control (N = 100): maltodextrin powder placebo	
Outcomes	NEC Death Invasive infection Length of hospital stay	
Notes	Setting: Turkey (5 centres: 2011 to 2014) Funding: not stated NB. This was a 4‐arm RCT‐ other groups were probiotics (N = 100) and synbiotics (N = 100)	
***Risk of bias***		
**Bias**	**Authors' judgement**	**Support for judgement**
Random sequence generation (selection bias)	Low risk	Computer‐generated sequence
Allocation concealment (selection bias)	Low risk	Sealed opaque envelopes
Blinding of participants and personnel (performance bias) All outcomes	Low risk	Masked (placebo‐controlled)
Blinding of outcome assessment (detection bias) All outcomes	Low risk	Masked (placebo‐controlled)
Incomplete outcome data (attrition bias) All outcomes	Low risk	Complete reporting
Selective reporting (reporting bias)	Low risk	Unlikely
Other bias	Low risk	No evidence of baseline imbalance

**Table:** Hascoët 2022

***Study characteristics***		
Methods	RCT	
Participants	86 preterm infants (27 to 32 weeks' gestation; and birth weight < 1700 g)*	
Interventions	Prebiotics (N = 43): 2′‐fucosyllactose (2′FL) and lacto‐N‐neotetraose (LNnT) in a 10:1 ratio (0.340 and 0.034 g/kg /day, respectively), given daily in three divided doses orally or via enteral tube from within 7 days of birth until discharge Control (N = 43): glucose placebo (0.14 g/kg /day)	
Outcomes	Time to full enteral feeds (150 mL/kg/day) Growth rates NEC Death Invasive infection Bronchopulmonary dysplasia Length of hospital stay	
Notes	Setting: France (7 hospitals; mid to late 2010s) Funding: Société des Produits Nestlé S.A. The funder was involved with interpretation of data, writing of the report, and the decision to submit it for publication. *Median gestation 30 weeks Further information courtesy of Prof Hascoët (chief investigator)	
***Risk of bias***		
**Bias**	**Authors' judgement**	**Support for judgement**
Random sequence generation (selection bias)	Low risk	Computer‐generated
Allocation concealment (selection bias)	Low risk	Computer‐allocated
Blinding of participants and personnel (performance bias) All outcomes	Low risk	Masked (placebo‐controlled)
Blinding of outcome assessment (detection bias) All outcomes	Low risk	Masked (placebo‐controlled)
Incomplete outcome data (attrition bias) All outcomes	Low risk	Complete reporting
Selective reporting (reporting bias)	Low risk	Unlikely
Other bias	Unclear risk	No evidence baseline imbalance. Funder (Nestlé S.A.) involvement with interpretation of data, writing of report, and decision to submit it for publication.

**Table:** Modi 2010

***Study characteristics***		
Methods	RCT	
Participants	160 preterm infants (< 33 weeks' gestation; "appropriately grown")*	
Interventions	Prebiotics (N = 77): GOS/FOS (9:1 mixture: 0.8 g/kg/day) in preterm formula daily until hospital discharge Control (N = 83): identical formula without prebiotic supplement NB. Preterm formula used only to augment insufficient maternal milk volume	
Outcomes	NEC Death Invasive infection Time to full enteral feeds (150 mL/kg/day) Length of hospital stay	
Notes	Setting: England (13 hospitals; mid‐late 2000s) Funding: Danone Research, Friedrichsdorf, Germany *Median gestation 30 weeks	
***Risk of bias***		
**Bias**	**Authors' judgement**	**Support for judgement**
Random sequence generation (selection bias)	Low risk	Computer‐generated
Allocation concealment (selection bias)	Low risk	Telephone‐randomisation
Blinding of participants and personnel (performance bias) All outcomes	Low risk	(150 mL/kg/day)
Blinding of outcome assessment (detection bias) All outcomes	Low risk	Masked (identical formulas)
Incomplete outcome data (attrition bias) All outcomes	Low risk	Near‐complete reporting (parental consent for 6 infants withdrawn postrandomisation)
Selective reporting (reporting bias)	Low risk	Unlikely
Other bias	Low risk	No evidence baseline imbalance

**Table:** Riskin 2010

***Study characteristics***		
Methods	RCT	
Participants	28 preterm infants (< 35 weeks' gestation)*	
Interventions	Prebiotics (N = 15): lactulose (1 g/100 mL) in all human milk or formula daily feeds until hospital discharge Control (N = 13): dextrose (1 g/100 mL)	
Outcomes	Stool colonisation patterns (probiotic bacteria) NEC Death Invasive infection Time to full enteral feeds Length of hospital stay	
Notes	Setting: Israel (2005 to 2006) Funding: Not stated *Mean gestation 29 weeks	
***Risk of bias***		
**Bias**	**Authors' judgement**	**Support for judgement**
Random sequence generation (selection bias)	Low risk	Pharmacy‐randomised
Allocation concealment (selection bias)	Low risk	Sealed non‐transparent envelopes
Blinding of participants and personnel (performance bias) All outcomes	Low risk	Masked (placebo‐controlled)
Blinding of outcome assessment (detection bias) All outcomes	Low risk	Masked (placebo‐controlled)
Incomplete outcome data (attrition bias) All outcomes	Low risk	Complete reporting
Selective reporting (reporting bias)	Low risk	Unlikely
Other bias	High risk	Mean gestational age and birth weight higher in intervention (30 weeks, 1523 g) than control group (28 weeks, 1270 g)

**Table:** van den Berg 2010

***Study characteristics***		
Methods	RCT	
Participants	113 very preterm or VLBW infants	
Interventions	Prebiotics (N = 55): GOS/FOS (80%) + pAOS (20%) (up to 1.5 g/kg/day) in all human milk or preterm formula feeds between days 3 and 30 after birth Control (N = 58): Maltodextrin (placebo)	
Outcomes	Invasive infection NEC Death Bronchopulmonary dysplasia Retinopathy of prematurity Neurodevelopment at corrected age of 2 years: Mental Development Index and Psychomotor Development Index of the Bayley Scales of Infant Development (2nd or 3rd edition) Cerebral palsy Visual impairment Auditory impairment	
Notes	Setting: Netherlands (2007 to 2008) Funding: Danone Research, Friedrichsdorf, Germany	
***Risk of bias***		
**Bias**	**Authors' judgement**	**Support for judgement**
Random sequence generation (selection bias)	Low risk	Computer‐generated sequence
Allocation concealment (selection bias)	Low risk	Computer‐allocated
Blinding of participants and personnel (performance bias) All outcomes	Low risk	Masked (placebo‐controlled)
Blinding of outcome assessment (detection bias) All outcomes	Low risk	Masked (placebo‐controlled)
Incomplete outcome data (attrition bias) All outcomes	Low risk	Complete reporting for in‐hospital outcomes [Neurodevelopmental outcomes at 2 years corrected available for 76% of eligible participants]
Selective reporting (reporting bias)	Low risk	Unlikely
Other bias	Low risk	No evidence baseline imbalance

cfu: colony‐forming units; FOS: fructo‐oligosaccharides; GOS: galacto‐oligosaccharides; pAOS: pectin‐derived acidic oligosaccharides; NEC: necrotising enterocolitis; RCT: randomised controlled trial; VLBW: very low birth weight

### Characteristics of excluded studies [ordered by study ID]

**Table:**

Study	Reason for exclusion
Dasopoulou 2015	Participants were late‐preterm (not very preterm) infants.
Fanaro 2005	Participants were term (not very preterm) infants.
Indrio 2009	Participants were late‐preterm (not very preterm) infants.
Kapiki 2007	Participants were term infants.
Luoto 2013	Participants were late‐preterm (not very preterm) infants.
Mihatsch 2006	Infants commenced supplements when fully formula‐fed (mean day 36 for prebiotics, and mean day 53 for maltodextrin placebo).
Neumer 2021	Participants were term infants.

## Differences between protocol and review

The protocol specified the population of interest as very preterm and VLBW infants in order to enhance applicability to those infants at high risk of developing NEC and associated complications. Some included trials included infants born up to 35 weeks' gestation. We included these trials provided most participants were very preterm and VLBW infants.
